# Cell-based regenerative joint therapy: a hot topic

**DOI:** 10.1007/s00167-022-06959-8

**Published:** 2022-04-01

**Authors:** P. Angele, P. Niemeyer, L. Laver, L. de Girolamo, J. Zellner

**Affiliations:** 1 Department of Trauma and Reconstructive Surgery, University Hospital Regensburg, Franz Josef Strauss Allee 11, 93042 Regensburg, Germany; 2 Sporthopaedicum Regensburg, Regensburg, Germany; 3 Orthopaedic Biotechnology Laboratory, IRCCS Istituto Ortopedico Galeazzi, 20161 Milan, Italy; 4 OCM Hospital for Orthopedic Surgery Munich, Munich, Germany; 5 Department of Orthopaedics, Hillel Yaffe Medical Center (HYMC), Hadera, Israel; 6 Rappaport Faculty of Medicine, Technion University (Israel Institute of Technology), Haifa, Israel

In the 90s, Tissue Engineering was introduced to repair biological tissue by application of cell- and/or biomaterial-based engineered constructs. The concept of tissue Engineering became popular through the “Vacanti mouse” [4], the mouse with the human ear on its back. The desire and hope to revolutionize important areas of medicine seemed to be fulfilled. However, despite 30 years of dedicated research, we are still relying on organ transplantation rather than growing organs in the lab. The question is—Which applications, produced by this technology, are in clinical use?

An excellent example for successful cell-based tissue regeneration has actually been developed in the field of orthopaedics, with the autologous chondrocyte transplantation, which was introduced in the 90s by Petersen and Brittberg [3]. With improvement of the technique by applying smart biomaterials, huge focal chondral defects in the knee joint can be successfully regenerated with autologous matrix-induced chondrocyte transplantation combined with a low failure rate [1]. The success can be scored by a significant improvement in clinical and radiological scores over long-term follow-up. Several prospective randomized trials have proven the potential and quality of this regenerative technique [7].

So, everything solved?

In our view, **cell-based regenerative joint therapy is actually still a very hot topic**.

- Autologous chondrocyte transplantation is a proven successful cell-based regenerative technique for the management of cartilage defects in the knee joint. But still, due to regulatory hurdles, this technique is not available in several European countries. Besides the knee joint, cartilage defects can be recognized in all other joints. Up to now, the clinical application of autologous chondrocyte transplantation is limited to the knee, ankle and the hip.
- According to the German Cartilage Registry the predominant proportion of cartilage defects (60%) are degenerative. An efficient regenerative treatment for degenerative lesions is still controversial because autologous chondrocyte transplantation is a technique which is introduced for traumatic cartilage defects. Recent studies show that also focal Early-OA cartilage defects can be treated successfully knowing that the failure rate is slightly higher than for isolated traumatic defects [1, 2].
- A variety of regenerative therapies, including intraarticularly applied hemo-components and cell-based therapies are currently in clinical development and are, in part, in clinical use. Such therapies face several practical opportunities and challenges and could reduce the health care burden, in part by replacing traditional drug therapies and highly invasive surgical interventions with smarter and less invasive treatments. Despite the increasing use of these so called “orthobiologics” (biological substances to help musculoskeletal injuries), and the consequent increase of the related literature, consistent and strong results about their efficacy are yet to come. This is mainly due to the lack of a consensus about indications and protocols, and in many cases these technologies are used indiscriminately. Moreover, therapy developers and providers must address hurdles from regulatory to reimbursement to commercial challenges before successful orthopedic cell therapies are available to patients.
- Mesenchymal stem cells (MSCs) have been proposed as an intriguing cell source to differentiated adult cells for repair and regeneration of different tissue structures of the joint like articular cartilage, meniscus, tendons and ligaments. MSCs might open the regeneration not only for cartilage defects but also the regeneration of other joint lesions to achieve a whole joint approach, which is important for a successful regenerative treatment outcome [6]. Due to safety concerns and the consequential regulatory burdens in many countries only a limited amount of stem cell based therapeutic strategies have been introduced in daily clinical practice up to now [9]. However, many preclinical studies have shown that MSCs can act like repair cells and differentiate into the target tissue to promote regeneration or provide bioactive substances that modulate the healing process [5]. The term medicinal signaling cells for MSCs highlights the many and various possibilities to support tissue regeneration like immunomodulation, antiinflammation, anti-apoptotic activity, neoangiogenesis, activation of resident stem cells, antimicrobial effects or selective migration ability (“homing”) [8]. The multilineage differentiation, the homing effect to various injured structures and the immunomodulating effect in a degenerative environment also illustrates the crucial role of MSCs in the future and the potential role of cell application for joint regeneration [9]. Research should now focus on the translation of this huge beneficial potential of MSCs in clinical application and show the safety of these techniques.

For this **hot topic “cell-based regenerative joint therapy”** a European strategy was established by ESSKA, called the “ESSKA Orthobiologics Initiative” (**ORBIT**), in order to address some of the issues mentioned earlier (regulatory, improve knowledge, improve indications, explore risk–benefit, cost–benefit and more) as well as achieve a meaningful consensus for therapies based on the use of orthobiologics. This includes the full spectrum of orthobiologic use, including “injectable” and “implantable” orthobiologics. Through several steps, starting from an internal survey onto generating and reaching a consensus on the use of orthobiologics for variable common indications, ORBIT aims to pave the way for further evolutions of these technologies and the identification of alternative financing and reimbursement models to support ongoing patient access and innovation.

Hot topics in the orthopaedic field need the input of our entire medical and scientific community. Only with combined efforts we will succeed. Therefore the “ESSKA Orthobiologics Initiative” (**ORBIT**) strives for close collaboration and support from ESSKA affiliated societies (e.g., DGOU, AGA) in the peer-review process of the consensus in generating a pan-European balanced voice. A good relationship of ESSKA, as a European-wide society, with strong societies and partners like AGA, Europe’s largest society for arthroscopy and joint therapy with over 5000 members, enables us to bring together clinicians and scientists and to share research, practical techniques and experience. Both societies, ESSKA and AGA, represent multiple countries and combine therefore a huge amount of knowledge especially in the regenerative joint treatment. This collaboration creates a win–win situation to strengthen the European perspective with the chance to join forces to progress the **hot topic “cell-based regenerative joint therapy”**.
